# Adolescents’ online communication and well-being: Findings from the 2018 health behavior in school-aged children (HBSC) study

**DOI:** 10.3389/fpsyt.2022.976404

**Published:** 2022-10-06

**Authors:** Nelli Lyyra, Niina Junttila, Jasmine Gustafsson, Henri Lahti, Leena Paakkari

**Affiliations:** ^1^Faculty of Sport and Health Sciences, University of Jyväskylä, Jyväskylä, Finland; ^2^Department of Teacher Education, University of Turku, Turku, Finland; ^3^Department of Teacher Education, University of Jyväskylä, Jyväskylä, Finland; ^4^Faculty of Social Sciences, University of Helsinki, Helsinki, Finland; ^5^Public Health Research Program, Folkhälsan Research Center, Helsinki, Finland

**Keywords:** online communication, well-being, health, loneliness, social media, adolescence

## Abstract

**Background:**

Digital transformation has influenced all areas of adolescents’ lives, including the ways adolescents maintain friendships. Interpersonal communication is one of the most common activities while online. Online communication may provide adolescents with opportunities to expand their social contacts, but these encounters can be risky, especially when the communication is with unknown people on the internet. This study examined the associations between different forms of online communication behavior and well-being.

**Materials and methods:**

Data were collected from Finnish adolescents as part of the Health Behavior in School-Aged Children (HBSC) study in 2018. The participants were 3,140 Finnish adolescents aged 11–15 years. Descriptive analyses were used to examine the frequency of different forms of online communication behaviors. The associations between online communications and individual factors were analyzed using the X^2^ test and 95% confidence intervals. Structural equation modeling (SEM) was used to analyze the extent to which adolescents’ online communication behavior explained the variance in adolescents’ well-being indicators.

**Results:**

Overall, 60% of the adolescents reported communicating intensively with close friends, with higher rates of intensive communication reported by girls, higher age groups, and the high health literacy group. 22% of adolescents reported intensive communication with friends they got to know through the internet (online friends), while intensive online communication with unknown people was reported by 13% of adolescents. Overall, around one-fourth of adolescents preferred sharing personal matters online rather than in face-to-face encounters, and 10% of adolescents reported using the internet daily to get to know new people, and to look for like-minded company. The SEM analysis showed that keeping online contact with offline friends was linked to a positive outcome in all the measured well-being indicators; however, intensive communication with people contacted only online (online friends and unknown people) was negatively associated with well-being indicators (lower self-rated health, lower life satisfaction, higher loneliness, and problematic social media use).

**Conclusion:**

Both positive and negative associations were observed between online communication and well-being, depending on the target and content of the communication. The results indicate that online communication has benefits for adolescents who have more offline social life. Overall, one should ensure that the impact of interventions is proportionately greater for adolescents at the bottom end of the health gradient.

## Introduction

Digital transformation has influenced all areas of adolescents’ lives (1, 2), including how they form and maintain friendships (3). Social media platforms have brought new opportunities to connect with other people, and interpersonal communication is one of the most common activities while online (4). On the internet, adolescents may interact with known people (e.g., friends) and also strangers. Some adolescents may even find online communication easier than face-to-face communication (5). Online activities may provide adolescents with opportunities to expand their social contacts, but these encounters can be risky, especially when the communication is with unknown people on the internet (6). This study aimed to complement current understandings of adolescents’ online communication behavior, and to clarify the association between different forms of online communication behaviors and well-being among adolescents.

Previous research has found both similarities and differences in social media use and online activities between girls and boys, and between young people from different age groups. In a review by Pujazon-Zazik and Park (7), adolescent girls and boys used the internet to a similar extent, but girls were more likely than boys to visit and/or have a social media profile, use instant messaging, create blogs, and post photographs. Furthermore, boys and older adolescents have been found to disclose personal information online (8) and to participate in excessive gaming on electronic devices more than girls (9). On the other hand, girls are more likely than boys to post “selfies” and content about their emotions and feelings, their family, and their religious beliefs (10), and to be excessive users of social media (9). According to the EU Kids Online 2020 report (5), almost one-third of European adolescents aged 9–16 reported that they often or always found it easier to be themselves online than when they were with people face-to-face, with this being more frequently reported by boys. Furthermore, regarding age differences, in most participating countries adolescents aged 14–16 spent almost twice as much time online as children aged 9–10, with older adolescents also being more likely to report a preference for talking online (rather than offline) about a range of matters (5). Another study found that older adolescents were more likely to interact with unknown people online than were younger adolescents (11).

As a consequence of adolescents’ increased social media use (5), concerns have been raised regarding its links with their well-being. Castellacci and Tveito (12) highlighted four mechanisms linking internet use to well-being, which were seen as having both positive and negative influences on well-being: the internet affects individuals’ time-use patterns, enables new activities (e.g., online games and digital social networks), provides easy access to information, and offers new communication tools. Use of social media may encompass positive aspects such as increased social support, reduced social anxiety, increased self-esteem, and decreased loneliness. Greater advantages have been reported by those young people who perceive their offline friendship quality to be high. On the other hand, potentially harmful aspects of social media use have also been noted, including increased exposure to depression and cyberbullying (13), and dissatisfaction with one’s personal appearance (14). Higher social media use has also been associated with lower self-esteem, higher levels of loneliness, and higher depression (15–18). Furthermore, greater engagement in online activities, with the addition of more personal details to social media profiles, has been associated with cyberbullying (19).

Intensive use of social media has also been linked to problematic social media use (20), that is, immoderate and obsessional use of social platforms (21). Problematic social media users are more likely to experience mental health problems such as depression, loneliness, and lower life satisfaction (22). The definition of problematic social media use is in line with the diagnostic addiction criteria for Internet gaming disorder; see also the latest version of the Diagnostic and Statistical Manual of Mental Disorders (DSM-5) (23). However, the phenomenon has not been formally acknowledged as a mental disorder.

Social media use may be more strongly related to well-being among certain groups of individuals, as measured for instance *via* gender or age, although the evidence is contradictory. For example, social media have been associated with poorer well-being only among girls (24), or with higher well-being among boys (25); by contrast, web communication has been linked to poorer well-being among boys, but not girls (26). Furthermore, the association between the use of social media platforms and well-being has been found to be positive among young adults but negative among older participants (27). Age differences in associations between social media use and well-being could potentially be explained by people’s life experiences with technology (28, 29), given that younger individuals have grown up using a wide variety of digital technologies. In systematic reviews, mixed evidence has been found for gender differences in the associations between children’s and adolescents’ social media use and depressive symptoms. A higher frequency of use, a tendency for social comparison, and higher investment in online communication were associated with depressive moods, especially among girls [e.g., (30)]. In addition, longitudinal evidence has indicated that higher social media engagement (in terms of commenting and liking others’ status updates and photographs) is linked to lower self-esteem among girls, but not boys (31). One potential explanation for these findings could be linked to ideals generated by social media since girls have been shown to internalize media body ideals to a greater extent and to feel more pressure from the media compared to boys (32). Finally, in a meta-analysis by Saiphoo et al. (18), neither gender nor age moderated associations between social networking site use and well-being in terms of lower self-esteem.

In studying the link between adolescents’ social media use and their well-being, an important question is also whether certain competencies (or their insufficiency) place adolescents in a vulnerable situation for lower well-being. Especially during the last decade, public health research has focused on health literacy as a set of competencies needed to function in different health contexts, including digital environments. Health literacy covers “personal knowledge and competencies that accumulate through daily activities, social interactions and across generations” and these “enable people to access, understand, appraise, and use information and services in ways that promote and maintain good health and well-being for themselves and those around them” (33). Health literacy is positively related to diverse health indicators among adolescents, including problematic social media use (34), self-rated health (34, 35), and life-satisfaction (36). There is also some evidence that among adults, higher eHealth literacy (i.e., involving both health literacy and the digital skills that enable people to use digital technologies for health purposes) is linked to better digital privacy protection skills (37). Among adolescents, being more concerned with personal online privacy is linked to a higher likelihood of adopting privacy protection behaviors such as removing personal information and blocking people on social media sites (38). In turn, individuals’ privacy settings have been related to the disclosure/non-disclosure of information online, such that the setting of Facebook profiles to “private” is associated with a lesser likelihood of disclosing personal information (i.e., insensitive and contact information) (8). Thus, it could be hypothesized that adolescents reporting low levels of health literacy would have a higher risk of sharing personal information online. To date, there has been no examination of whether adolescents reporting different levels of health literacy differ in their online communication behaviors, or whether potential associations between adolescents’ online activities and well-being differ depending on their levels of health literacy.

Because social media platforms and online communication tools are constantly changing, there is a need for a more detailed analysis of adolescents’ engagement in specific online communication behaviors. As noted above, there may be both benefits and disadvantages in adolescents’ social media use and online activities. Previous studies examining the associations between adolescents’ online behaviors and well-being have contained mostly limited information on the quality of online communication, whereas the present study collected data on several types of online communication behaviors and indicators of well-being. The current study also adds to the existing literature by examining differences in online communication behaviors and their links to well-being by gender, age, and level of health literacy, thus allowing possibilities to identify vulnerable subgroups of adolescents.

### Aim of this study

This study aimed to complement current understandings of adolescents’ online communication behavior, and to shed light on the association between different forms of online communication behaviors and well-being among adolescents. The research questions were as follows:

1. How prevalent among adolescents are different forms of online communication behaviors (intensive online communication, a preference for online communication on personal matters over face-to-face encounters, and use of the internet daily to look for company), and how are these associated with individual factors (gender, age, or the level of health literacy)?
2. What associations exist between different forms of online communication and well-being indicators (self-rated health, life satisfaction, loneliness, problematic social media use, and cyberbully victimization)?
3. Do associations between online communication behaviors and well-being indicators differ by individual factors?

## Materials and methods

### Study design

Data were collected from Finnish adolescents as part of the Health Behavior in School-Aged Children (HBSC) study in 2018. The HBSC study is an international World Health Organization collaborative study using cross-sectional surveys performed every fourth year among students aged 11, 13, and 15 years. To ensure a nationally representative sample of the target population, the sample was taken from the Finnish school register *via* a cluster sampling method, following the international HBSC protocol (39, 40). The primary sampling unit was the school, and within each school one class was randomly selected. The school-level response rate was 57%. Participation was voluntary, and students completed a standardized questionnaire during a school lesson, following instructions from a teacher. Students responded anonymously and completed the web-based questionnaire *via* Webropol software (Webropol Oy, Helsinki, Finland). The Ethics Committee of the University of Jyväskylä approved ethical issues.

### Participants

The participants were 3,140 Finnish adolescents aged 11 years (*n* = 946), 13 years (*n* = 1,118), and 15 years (*n* = 1,076). The sample included a similar number of boys (*n* = 1,557) and girls (*n* = 1,594). Age and gender were not associated in the sample [χ^2^(2) = 0.177, *p* = 0.915], meaning that a similar proportion of boys and girls were in each age group.

### Measures

#### Individual factors

Students were asked to self-report their age and gender (1 = Boy, 2 = Girl).

Health Literacy (HL) was measured by the Health Literacy for School-Aged Children (HLSAC) instrument (36, 41), which includes ten items measuring five core competencies to make health-related decisions (theoretical knowledge, practical knowledge, critical thinking, self-awareness, and citizenship). Students were asked to assess each item on a four-point scale (1 = Not at all true, 2 = Not completely true, 3 = Somewhat true, 4 = Absolutely true). Responses were recoded into three categories (HL levels) based on a scale sum score (Low = sum score 10–25, Moderate = sum score 26–25, High = sum score 36–40). Cronbach’s alpha was 0.96.

#### Online communication behavior

The intensity of online communication was measured by four items adapted from the EU Kids Online Survey (42) asking how often respondents had online contact with (i) “Close friend(s),” (ii) “Friends from a larger friend group,” (iii) “Friends that you got to know through the internet but didn’t know before” (online friends), (iv) “Unknown people” (Finnish national item). The frequency of communication was assessed by five response options (1 = Never or almost never, 2 = At least every week, 3 = Daily or almost daily, 4 = Several times each day, 5 = Almost all the time throughout the day). The response options “Several times each day” and “Almost all the time throughout the day” were combined to indicate intensive online communication. Following structural equation modeling items “Close friends” and “Friends from a larger friend group” were combined to encompass online communication with friends who were also met offline. The items “Friends that you got to know through the internet but didn’t know before” and “Unknown people” were combined to encompass online communication with persons one communicates with only online (see also section “Statistical analysis”). Cronbach’s alpha was 0.75.

Preference for online communication in personal matters was measured by three items asking students their opinion about disclosing personal information: “On the internet, I talk more easily about… (i) secrets, (ii) my inner feelings, (iii) concerns than in a face-to-face encounter”; (43). The response options were: 1 = Strongly disagree, 2 = Disagree, 3 = Neither agree nor disagree, 4 = Agree, 5 = Strongly agree. The response options “Agree” and “Strongly agree” were combined to indicate a preference for online communication in personal matters. Cronbach’s alpha was 0.92.

Company-seeking behavior on the Internet was measured by two items from the Internet activity scale (44): “Getting to know new people,” and “Looking for like-minded company.” Adolescents responded with six options (1 = Never, 2 = Less than once a week, 3 = Once a week, 4 = Several days a week, 5 = Every day once a day, 6 = Several times every day). The response options “Every day once a day” and “Several times every day” were combined to indicate daily internet activity. Cronbach’s alpha was 0.85.

#### Well-being indicators

Self-rated health (SRH) was evaluated by a single question measuring the individual’s perception and evaluation of their health (45) *via* four response options (4 = Excellent, 3 = Good, 2 = Fair, 1 = Poor). For the analysis, the scale was reverse-scored.

Life satisfaction, also known as Cantril’s Ladder, is a simple visual scale to assess general life-satisfaction (46). The ladder consists of ten steps. Step 10 on top represents the best possible life situation, while bottom step 0 represents the worst possible situation.

Perceived loneliness was assessed using a single question on global loneliness: “Do you ever feel lonely?” with four response options (1 = No, 2 = Yes, sometimes, 3 = Yes, quite often, 4 = Yes, very often). The question on global perceived loneliness is included in the Finnish national HBSC questionnaire.

Problematic social media use (PSMU) was measured with nine items (47) using dichotomous (No/Yes) response options. The items covered the following dimensions: preoccupation, tolerance, withdrawal, displacement, escape, problems, deception, displacement, and conflict. The cut-off value for the problematic use group was 6 or more “yes” answers (34, 48); for the moderate risk group 2–5 “yes” answers, and for the no-risk group 0–1 “yes” answers (34). Cronbach’s alpha was 0.83.

Cyberbullying victimization applies a one-item measure that assesses the frequency of being bullied online over the past 2 months (49). The question has five response options (1 = Haven’t, 2 = Once or twice, 3 = Two to three times per month, 4 = Once a week, 5 = Several times a week).

### Statistical analysis

Descriptive analyses were used to examine the frequency of different forms of online communication behaviors (intensive online communication, preference for online communication, company-seeking behavior) in the total sample and within subgroups based on individual factors (gender, age, and health literacy). The associations between grouping variables and digital communication behavior were analyzed using the X^2^ test and 95% confidence intervals. All the descriptive analyses were conducted by Stata (version 16).

Structural equation modeling was used to analyze the extent to which adolescents’ online communication behavior explained the variance in adolescents’ well-being indicators. First, the structure of online communication behavior was analyzed, arriving at a four-factor model. Factor 1 encompassed Intensive online communication with close friend(s) and larger friend groups, Factor 2 Intensive online communication with persons who were communicated with only online (online friends and unknown people), Factor 3 A preference for sharing personal content online, and Factor 4 Company-seeking behavior. This was done separately for the total sample, and for each subgroup formed by gender, age, and HL.

Secondly, structural equation modeling was used to determine the extent to which the different online communication factors explained the variance in the well-being indicators (self-rated health, life-satisfaction, loneliness, problematic social media use, and cyberbullying victimization). This was done by estimating the paths (regression coefficients) from the four latent factors (measuring online communication behavior) to the five well-being indicators.

Multigroup invariance analysis was used to analyze gender, age, and HL level differences in factor structure and regression coefficients by estimating models simultaneously for each subgroup (configural invariance, M1), and by adding constraints to the factor loadings (metric invariance, M2) and regression coefficients (scalar invariance, M3). The model fit was evaluated using the Chi-square test, the root mean square error of approximation (RMSEA), the comparative fit index (CFI), the Tucker–Lewis index (TLI), and the standardized root mean square residual (SRMR). As Chi-square is highly sensitive to large sample sizes, relative measures for the goodness of fit are recommended in addition to the Chi-square test (50). The following cut-off values were used: RMSEA < 0.06; SRMR < 0.08; CFI > 0.95; TLI > 0.95 (51). The goodness of fit in the invariance testing was analyzed by ΔCFI and ΔRMSEA, with a difference of less than –0.010 in CFI and 0.015 in RMSEA indicating that the nested models had an equal factor structure (M1, M2) and a similar strength in regression coefficients (M2, M3) (52).

The parameters were estimated using the maximum likelihood robust (MLR) estimator and the missing-at-random (MAR) data procedure. The analyses were conducted using Mplus 7.0 (53).

## Results

### Prevalence of different forms of online communication behavior and associations with individual factors (RQ1)

Overall, 60% of the adolescents reported communicating intensively with close friends, with higher rates of intensive communication being reported by girls (girls 69.0%, boys 49.1%, *p* < 0.001), higher age groups (15 years 70.8%, 13 years 63.5%, 11 years 41.1%, *p* < 0.001), and the high health literacy group (high HL 73.1%, moderate HL 64.7%, low HL 59.9%, *p* < 0.001) (Table 1). Moreover, it was more common for boys than for girls to communicate only rarely with close friends (boys 5.4%, girls 1.7%, Supplementary Table 1). Intensive communication with larger friend groups was more prevalent among higher age groups (13- and 15-year-olds, 36.0 and 42.3% respectively) than among 11-year-olds (23.8%, *p* < 0.001), and more prevalent among adolescents in the high health literacy group (44.9%) than among those in the moderate (36.7%) or low health literacy groups (30.8%, *p* < 0.001).

**TABLE 1:** Prevalence of intensive online communication, preference for online communication, and daily internet use to look for new friends and company.

		Gender			Age				Health literacy			
	All %	Boys % [95% CI]	Girls % [95% CI]	*P*-value	11-year % [95% CI]	13-year % [95% CI]	15-year % [95% CI]	*P*-value	Low % [95% CI]	Moderate % [95% CI]	High % [95% CI]	*P*-value
**Intensive online communication^a^**												
Close friends	59.5 [56.4–62.5]	49.1 [45.0–53.3]	69.0 [65.5–72.3]	< 0.001	41.4 [37.5–45.4]	63.5 [59.1–67.7]	70.8 [67.8–73.6]	< 0.001	59.9 [50.0–69.0]	64.7 [61.2–68.1]	73.1 [69.3–76.7]	< 0.001
Larger friend group	34.6 [32.4–36.9]	34.3 [31.2–37.6]	34.9 [32.1–37.9]	0.945	23.8 [20.6–27.2]	36.0 [32.5–39.6]	42.3 [39.1–45.5]	< 0.001	30.8 [23.7–38.9]	36.7 [33.5–40.1]	44.9 [40.8–49.1]	< 0.001
Online friends	22.4 [20.5–24.3]	21.7 [19.1–24.6]	23.0 [20.7–25.5]	0.666	13.8 [10.9–17.2]	22.5 [19.8–25.4]	27.7 [25.1–30.5]	< 0.001	29.7 [21.5–39.4]	23.9 [21.2–26.7]	26.3 [22.9–30.1]	0.470
Unknown people	12.9 [11.2–14.7]	16.1 [13.7–18.8]	9.5 [7.5–12.0]	< 0.001	8.5 [6.0–12.0]	14.3 [11.5–17.6]	13.9 [11.4–16.9]	< 0.001	15.8 [9.3–25.6]	12.1 [10.0–14.7]	16.4 [13.1–20.3]	0.018
**Preference for online communication in personal matters^b^**												
Secrets	21.9 [20.1–23.7]	23.5 [21.2–26.0]	20.3 [18.1–22.7]	0.099	13.3 [11.1–15.9]	24.0 [21.6–26.7]	27.0 [24.2–29.9]	< 0.001	32.9 [26.6–39.9]	25.6 [23.1–28.2]	23.3 [20.0–26.8]	< 0.001
Feelings	27.7 [25.8–29.7]	24.3 [22.0–26.7]	30.9 [28.2–33.7]	< 0.001	17.3 [14.8–20.1]	31.2 [28.5–34.1]	32.3 [29.7–36.4]	< 0.001	36.5 [29.0–44.8]	33.1 [30.0–36.3]	29.0 [25.4–32.9]	< 0.001
Concerns	23.3 [21.5–25.2]	20.6 [18.4–23.0]	25.8 [23.3–28.4]	0.004	13.5 [11.1–16.3]	25.5 [23.2–27.9]	29.6 [26.8–32.5]	< 0.001	34.1 [27.9–40.9]	27.4 [24.6–30.3]	25.7 [22.5–29.2]	< 0.001
**Company-seeking behavior on the internet^c^**												
Get to know new people	10.0 [8.8–11.3]	12.9 [11.0–15.1]	7.3 [6.1–8.8]	< 0.001	6.4 [5.0–8.1]	10.8 [9.1–12.8]	12.2 [10.0–14.7]	< 0.001	12.0 [7.4–18.9]	10.0 [8.2–12.2]	13.4 [10.9–16.3]	0.120
Looking for like-minded company	9.5 [8.4–10.6]	11.6 [9.9–13.5]	7.5 [6.2–9.0]	< 0.001	6.8 [5.4–8.4]	9.9 [8.3–11.6]	11.3 [9.3–13.8]	< 0.001	13.1 [8.2–20.4]	9.4 [7.9–11.1]	12.0 [9.5–15.0]	0.394

Associations (X^2^) and differences (95% CI) by gender, age, and health literacy.

^a^ Categories: “Several times a day”. “All the time throughout the day”.

^b^ Categories: “Strongly agree”. “Agree”.

^c^ Categories: “Every day once a day”; “Several times every day”.

According to our analysis, 22% of adolescents reported intensive communication with friends they got to know through internet (online friends), this being more common among older age groups (Table 1). Intensive online communication with unknown people was reported by 13% of adolescents, with a higher prevalence among boys (boys 16.1%, girls 9.5%, *p* < 0.001). Though age differences could not be found regarding intensive communication, a higher proportion of 11-year-olds (71%) reported being “rarely” in contact with unknown people than was the case among 13- and 15-year-olds (48% and 53% respectively) (Supplementary Table 2). Similarly, in relation to health literacy, despite no differences in intensive communication, a larger proportion of persons with moderate health literacy (51%) reported “rarely” communicating with unknown people than was the case among those with low health literacy (38%) (Supplementary Table 3).

Overall, around one-fourth of adolescents preferred sharing personal matters online rather than in face-to-face encounters. Girls preferred sharing feelings (girls 30.9%, boys 24.3%) and concerns (girls 25.8%, boys 20.6%) more frequently. Furthermore, older adolescents (13- and 15-year-olds) preferred online communication more frequently than did 11-year-olds (e.g., regarding secrets: 11 years 13.3%, 15 years 27.0%, *p* < 0.001). Persons with higher health literacy were more likely to report not preferring to share feelings, concerns, or secrets online (Supplementary Table 3).

In the total sample, 10% of adolescents reported using the internet daily to get to know new people, and to look for like-minded company, boys more often than girls (boys 12.9%, girls 7.3%, *p* < 0.001). Furthermore, the prevalence of using the internet for getting to know new people was higher among older adolescents, and 15-year-olds looked for like-minded company online more frequently than did 11-year-olds (15 years 11.3%, 11 years 6.8%) (Table 1).

### Associations between online communication behavior and well-being indicators (RQ2)

**FIGURE 1:**
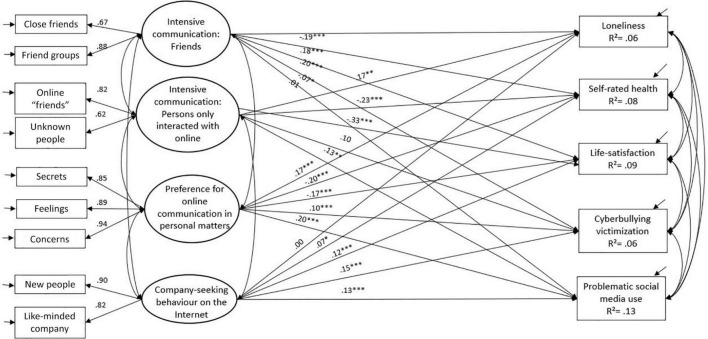
Association between different forms of online communication behavior and well-being indicators. Standardized beta coefficients are reported (*p < 0.05, ^**^p < 0.01, ^***^p < 0.001). Correlations are presented in Supplementary Table 4.

A structural equation model with four online communication latent variables explaining the variance of five well-being indicators (self-rated health, life satisfaction, loneliness, PSMU, cyberbully victimization) showed an excellent fit [X^2^(46) = 187.25; CFI = 0.99; TLI = 0.98; RMSEA = 0.03, SRMR = 0.02] (Figure 1).

Intensive online communication with friends was associated with higher life satisfaction (0.20, *p* < 0.001), better self-rated health (0.18, *p* < 0.001), and lower levels of loneliness (−0.19, *p* < 0.001). Intensive communication with friends was not associated with problematic social media use, and was negatively associated with cyberbullying victimization (−0.07, *p* = 0.043). Moreover, company-seeking behavior on the internet was positively associated with life satisfaction (0.12, *p* < 0.001) and self-rated health (0.07, *p* < 0.01), but also with higher rates of problematic social media use (0.13, *p* < 0.001) and being cyberbullied (0.15, *p* < 0.001) (Figure 1).

Intensive communication with persons who were only interacted with online (online friends, unknown people) was associated with lower life satisfaction (−0.33, *p* < 0.001), poorer self-rated health (−0.23, *p* < 0.001), experiencing loneliness more often (0.17, *p* = 0.003), and problematic social media use (0.15, *p* = 0.008). Preferring online communication to share personal matters such as feelings and secrets was associated with lower life satisfaction (0.17, *p* < 0.001), poorer self-rated health (−0.20, *p* < 0.001), higher rates of loneliness (0.17, *p* < 0.001), problematic social media use (0.20, *p* < 0.001), and being cyberbullied (0.10, *p* < 0.001) (Figure 1).

### Multi-group invariance and associations between different forms of online communication and well-being indicators by gender, age, and health literacy (RQ3)

Multi-group comparisons were used to analyze how strongly online communication explained the variation in well-being indicators according to gender, age, and health literacy. Using a multi-group model, the model was estimated simultaneously for all subgroups. This was done firstly with no constraints in factor loadings (model M1), and secondly by setting similar factor loadings (model M2). The results of model comparisons (ΔCFI values between 0.000 and 0.002; ΔRMSEA values between 0.000 and 0.002) indicated that the factor loadings were the same across gender, age, and health literacy subgroups. Furthermore, the model comparisons between models M2 and M3 (ΔCFI values between 0.002 and 0.004; ΔRMSEA value 0.002 for gender, age, and HL invariance) indicated that regression coefficients could be set as being equal across subgroups, meaning that the strength with which online communication variables explained the variance in well-being indicators was similar across gender, age, and level of health literacy (Table 2).

**TABLE 2:** Multi-group invariance and model comparisons across gender, age, and health literacy.

	Model fit indices						Model comparison	
Model	X^2^(df)	*P*	CFI	TLI	RMSEA	SRMR	Δ CFI	Δ RMSEA
**Gender invariance**								
M1 gender	184.06 (92)	<0.001	0.992	0.984	0.025	0.020		
M2 gender	211.10 (97)	<0.001	0.990	0.981	0.027	0.023	0.002	0.002
M3 gender	268.79 (117)	<0.001	0.986	0.979	0.029	0.034	0.004	0.002
**Age invariance**								
M1 age	266.00 (138)	<0.001	0.988	0.976	0.030	0.023		
M2 age	276.87 (148)	<0.001	0.988	0.978	0.029	0.025	0.000	0.001
M3 age	334.74 (188)	<0.001	0.986	0.980	0.027	0.320	0.002	0.002
**Health literacy invariance**								
M1 HL	287.61 (138)	<0.001	0.980	0.961	0.040	0.280		
M2 HL	312.25 (148)	<0.001	0.978	0.960	0.040	0.029	0.002	0.000
M3 HL	370.93 (188)	<0.001	0.976	0.965	0.038	0.036	0.002	0.002

M1, no restrictions, configural invariance; M2, equal factor loadings, metric invariance; M3, equal factor loadings and equal regression coefficients, scalar invariance.

Regression coefficients between the online communication variables and well-being indicators are presented in Table 3 for all subgroups. Lower levels of loneliness were explained by intensive online communication with friends (β varied from −0.06 to −0.31), while intensive online communication with persons who were only interacted with online appeared to demonstrate the opposite effect, being associated with higher rates of loneliness (β varied from 0.05 to 0.35). Furthermore, a preference for online communication in personal matters was associated with a higher degree of loneliness. The association between intensive online communication and loneliness was especially strong among girls (intensive communication with friends: β = −0.31; intensive communication with persons only interacted with online: β = 0.35); altogether, 14% of the variance in loneliness was explained by the four online communication variables.

**TABLE 3:** Regression coefficients between different forms of online communication and well-being indicators in subgroups formed by gender, age, and health literacy.

	Gender		Age			Health literacy		
	Boy β (p)	Girl β	11 years β	13 years β	15 years β	Low β	Mod β	High β
**Loneliness**	(*R*^2^ = 0.04)	(*R*^2^ = 0.14)	(*R*^2^ = 0.10)	(*R*^2^ = 0.04)	(*R*^2^ = 0.08)	(*R*^2^ = 0.07)	(*R*^2^ = 0.04)	(*R*^2^ = 0.03)
Intensive communication: Friends	−0.16**	−0.31***	−0.30***	−0.06	−0.26***	−0.10	−0.12*	−0.08
Intensive communication: People one communicates with only online	0.05	0.35***	0.25*	0.09	0.13	0.19	0.06	0.06
Preference for online communication in personal matters	0.10**	0.18***	0.16***	0.17***	0.17***	0.05	0.17***	0.16**
Company seeking behavior on the internet	0.11*	−0.03	0.03	−0.03	0.02	0.14	0.03	−0.02
**Self-rated health**	(*R*^2^ = 0.06)	(*R*^2^ = 0.11)	(*R*^2^ = 0.09)	(*R*^2^ = 0.07)	(*R*^2^ = 0.06)	(*R*^2^ = 0.08)	(*R*^2^ = 0.05)	(*R*^2^ = 0.03)
Intensive communication: Friends	0.17***	0.22***	0.20**	0.19**	0.19***	0.28*	0.09	0.11
Intensive communication: People one communicates with only online	−0.12*	−0.36***	−0.25*	−0.30**	−0.09	−0.24	−0.20**	−0.04
Preference for online communication in personal matters	−0.21***	−0.15***	−0.24***	−0.14***	−0.16***	−0.09	−0.13***	−0.11*
Company seeking behavior on the internet	0.03	0.09	0.13	0.16**	−0.03	−0.04	0.13*	0.08
**Life-satisfaction**	(*R*^2^ = 0.04)	(*R*^2^ = 0.17)	(*R*^2^ = 0.13)	(*R*^2^ = 0.07)	(*R*^2^ = 0.08)	(*R*^2^ = 0.17)	(*R*^2^ = 0.03)	(*R*^2^ = 0.05)
Intensive communication: Friends	0.12*	0.32***	0.30***	0.16*	0.19**	0.19	0.09	0.12
Intensive communication: People one communicates with only online	−0.16*	−0.55***	−0.41**	−0.32**	−0.23**	−0.45*	−0.19*	−0.23*
Preference for online communication in personal matters	−0.15***	−0.12**	−0.17**	−0.11**	−0.18***	−0.25**	−0.10**	−0.15**
Company seeking behavior on the internet	0.08	0.14*	0.11	0.20**	0.07	0.26*	0.09	0.09
**Cyberbullying victimization**	(*R*^2^ = 0.03)	(*R*^2^ = 0.10)	(*R*^2^ = 0.11)	(*R*^2^ = 0.05)	(*R*^2^ = 0.07)	(*R*^2^ = 0.07)	(*R*^2^ = 0.05)	(*R*^2^ = 0.08)
Intensive communication: Friends	−0.07	−0.08	0.02	−0.07	−0.10	−0.19	0.01	−0.14*
Intensive communication: People one communicates with only online	0.08	0.11	0.06	0.15	0.10	0.20	0.06	0.15
Preference for online communication in personal matters	0.10**	0.08*	0.12**	0.14**	0.06	0.03	0.07*	0.15***
Company seeking behavior on the internet	0.10	0.23***	0.24***	0.02	0.19***	0.11	0.14*	0.09
**Problematic social media use**	(*R*^2^ = 0.11)	(*R*^2^ = 0.19)	(*R*^2^ = 0.11)	(*R*^2^ = 0.12)	(*R*^2^ = 0.17)	(*R*^2^ = 0.14)	(*R*^2^ = 0.13)	(*R*^2^ = 0.16)
Intensive communication: Friends	−0.01	0.01	0.02	−0.01	0.03	−0.14	0.08	0.04
Intensive communication: People one communicates with only online	0.08	0.20*	0.06	0.23**	0.09	0.14	0.18*	0.09
Preference for online communication in personal matters	0.16***	0.20***	0.24***	0.18***	0.19***	0.23**	0.10**	0.24***
Company seeking behavior on the internet	0.19***	0.13*	0.11	0.03	0.24***	0.15	0.12*	0.16*

Factor loadings are set equal, standardized beta coefficient estimates (β), and R^2^ values are reported.

*p < 0.05, **p < 0.01**, ***p < 0.001.

Self-rated health and life satisfaction were associated similarly with the online communication variables. Intensive communication with friends explained higher SRH (β varied from 0.09 to 0.28) and life satisfaction (β varied from 0.09 to 0.32). A negative effect on SRH emerged in respect of intensive communication with persons who were interacted only online (SRH: β varied from −0.04 to −0.36; life satisfaction: β varied from −0.19 to −0.55) and a preference for online communication (SRH: β varied from −0.09 to −0.24; life satisfaction: β varied from −0.10 to −0.25). Online communication variables explained the highest amount of variance in SRH among girls (*R*^2^ = 0.17). Among girls, and in the low HL group, 17% of the variance in life satisfaction was explained by the online communication variables.

Cyberbullying victimization was explained by a preference for sharing personal matters online among both genders, among 13-and 15-year-olds, and in the moderate and high HL groups (β varied from 0.07 to 0.15). Using the internet daily for getting to know new people and like-minded company was associated with more frequent cyberbullying victimization among girls (β = 0.23), among 11- and 15-year-olds (β = 0.24, β = 0.19), and in the moderate HL group (β = 0.14). The highest R^2^ value was observed among 11-year-olds: 11% of the variance in cyberbullying victimization was explained by the online communication variables.

Problematic social media use was associated with a preference for online communication in personal matters in all the subgroups (β varied from 0.16 to 0.24). Company-seeking behavior was associated with PSMU among boys (β = 0.19) and girls (β = 13), among 15-year-olds (β = 0.24), and in the moderate (β = 0.12) and high (β = 0.16) HL groups. The highest amount of variance explained by the online communication variables in respect of PSMU was found among girls (*R*^2^ = 0.19).

## Discussion

Digital transformation has influenced all areas of adolescents’ lives, as young people have been among the first large-scale adopters of digital communication technologies (1, 2). The present study investigated associations between adolescents’ online communication and well-being using nationally representative cross-sectional data. The results indicate that adolescents’ online communication explains the variance in both positive and negative well-being, depending on the communication partner (see also (54) and the purpose of the communication. In general, the majority benefited from online communication, while a minor proportion experienced negative effects.

The findings of this study showed that keeping online contact with offline friends was linked to a positive outcome in all the measured well-being indicators, whereas intensive communication with people one communicates with only online was associated with negative well-being indicators (lower self-rated health, lower life satisfaction, loneliness, problematic social media use). This would indicate that online communication comes with benefits for those who already have offline friends, and who are accepted by peers and/or classmates. Online friendships are often considered to be weak, infrequent, superficial, and easily broken [e.g., (55, 56)], and they may not last long enough to offer intimacy and support (57). Hence, relying merely on online friends may contribute to a depressive mood (58), and also to greater experiences of loneliness and decreased life satisfaction, especially among girls and younger age groups, as found in this study. However, online friendships may serve as important means to relieve other well-being indicators not measured in this study, such as social anxiety (59) or identity development (60).

In our results, the majority of adolescents (60%) reported intensive online communication with close friends and one-third reported intensive communication with a larger friend group. Intensive online communication with close friends was more frequently reported by girls, older adolescents, and those in the high health literacy group. Intensive online communication with larger friend groups was more prevalent among older adolescents, and also among those in the high health literacy group. Intensive online communication with existing offline friends (“Close friends”) and friend groups (“Friends from a larger friend group”) gave indications of being beneficial to adolescent well-being. For example, online contact with friends and friend groups was associated with higher self-rated health and life satisfaction, in parallel with less loneliness and cyberbullying victimization. Studies on adolescent social behavior [e.g., (61)] indicate that the core qualities and components of adolescents’ face-to-face interactions (including interactivity, social reward, social support, and information disclosure) remain present when they communicate online. Such findings reinforce the notion that online interaction may have the function of complementing face-to-face encounters. Thus in the period of adolescence—which is hypersensitive to social stimuli in terms of well-being (62)—online communication with existing friend groups may enhance natural social processes and support well-being. Moreover, adolescents who intensively use online spaces to communicate with friends report online communication as helping them to understand their friends’ feelings; they thus feel more connected to their friends, with positive effects on the quality and closeness of the friendship (62). Consequently, young people with good existing friendships outside of online spaces would seem to be the persons who benefit most from the opportunities provided by digital communication. However, further research is needed to examine whether and how different online and offline assets accumulate in such a way as to bring greater health and well-being benefits [see (63)].

For adolescents with poor social skills, talking with strangers may compensate for difficult online social interactions (60). Furthermore, online communication on sensitive issues may be experienced as more comfortable (57). In this study, approximately one-eighth of the adolescents reported communicating intensively with unknown people and one-fifth with “friends” they had only met online. Intensive online communication with unknown people was more prevalent among boys, and intensive communication with online “friends” among older adolescents. Furthermore, one-fourth of the adolescents preferred talking about concerns and feelings online and one-fifth preferred talking online about secrets. The prevalences were higher in the older age groups, and sharing feelings and concerns was more prevalent for girls than for boys (see also 8, 10). Adolescents with higher health literacy were less likely to prefer sharing feelings, concerns, or secrets online. Despite many positive elements, our findings suggested that a preference for sharing personal content online and communication with online friends (and strangers) can place adolescents into a vulnerable situation in terms of loneliness (64), cyberbullying (65), problematic social media use, poor self-rated health, and low life-satisfaction. Note, however, that loneliness may also serve as a predisposing factor to sharing personal issues online (60). A major concern regarding interaction with online strangers and a preference for sharing personal content online is the possibility that children or adolescents will be sexually victimized (66).

The present study has several strengths. For instance, we utilized a large nationally representative database and validated instruments and made a conceptual distinction between different forms of digital communication. Note, however, that although this study was based on a nationally representative sample of 11-, 13-, and 15-year-old Finnish adolescents, caution would be needed in terms of generalizing the findings to (for instance) non-White and low-income countries. Moreover, since the data date back to 2018, it does not fully reflect adolescents’ current ways of communicating online. Several other study limitations should also be acknowledged. The cross-sectional design of our study did not allow for causal inferences. Hence, the present study could not determine with certainty whether the outcomes observed were an effect of digital communication or, for instance, of latent predispositions, personality traits, or social factors. Moreover, all the measures were based on self-report instruments, which may be susceptible to bias. Parry et al. (67) have concluded that self-report measures of media use, quantity, or duration should not be regarded as a valid stand-in for more objective measures. For the future, we would therefore suggest a need for longitudinal research to verify the direction of the associations between digital communication and well-being. Moreover, objective measurements of digital communication (including social media applications) should be used to verify the intensity of digital communication behavior.

To conclude, online communication variables explained the variation in measured well-being indicators in the total sample, ranging from 6% (loneliness and cyberbullying victimization) to 13% (problematic social media use). In addition, a group-level examination showed variation ranging from 3% (e.g., in relation to cyberbullying victimization among boys) to 19% (in relation to problematic social media use among girls). Here a crucial question is how large the coefficient of determination should be if one is seeking to define a factor as a critical determinant of adolescents’ well-being, to the extent of requiring intervention. In line with our earlier discussion on the role of health literacy in addressing health disparities (68), we would claim that “all factors that contribute to decreasing the disparities in health [and well-being] are important,” including online communication. In this regard, is worth noting that (for example) self-rated health has been found to predict mortality (69).

Our results further indicate that in addressing adolescent digital communication one would in the best case try to ensure that the impact of interventions is proportionately greater for adolescents who are in vulnerable situations, in terms of negative well-being associated with social media use (70). Thus, to address the association between intensive communication with unknown people and lower life satisfaction, one might wish to target actions at girls, younger age groups, and persons with low health literacy. However, given that the “girl” status explains more of the association between intensive communication with friends/friend groups and higher life satisfaction, a focus merely on girls in social media communication interventions might have the unwelcome result of increasing the disparities between girls and boys. All in all, it will be important to take into account person-specific effects in research, prevention, and intervention programs (71).

## Data availability statement

The raw data supporting the conclusions of this article will be made available by the authors, without undue reservation.

## Ethics statement

The studies involving human participants were reviewed and approved by the University of Jyväskylä. Written informed consent from the participants’ legal guardian/next of kin was not required to participate in this study in accordance with the national legislation and the institutional requirements.

## Author contributions

LP, NL, HL, and NJ: conceptualization. NL, NJ, and LP: methodology. NL and NJ: formal analysis. LP, HL, JG, and NL: investigation and writing (preparation of the original draft). JG, HL, LP, NJ, and NL: reviewing and editing. NL: visualization. LP: funding acquisition and resources. All authors contributed to the article and approved the submitted version.
