# Supplementary material for: Adolescents’ online communication and well-being: Findings from the 2018 health behavior in school-aged children (HBSC) study
Source: Front Psychiatry. 2022 Oct 6;13:976404. doi: 10.3389/fpsyt.2022.976404 (PMC9583151; doi:10.3389/fpsyt.2022.976404)
Supplement: Supplementary file 1 [file Table_1.DOCX]

Supplement 1. Prevalence of intensive online communication, preference for online communication, and using the internet daily to look for new friends and company. Associations (Χ²) and differences (95% CI) by **gender**.

|  | All  % [95% CI] | Boys  % [95% CI] | Girls  % [95% CI] | Χ²(df); p-value |
| --- | --- | --- | --- | --- |
| **Intensive online communication** |  |  |  |  |
| Close friends |  |  |  | 129.8(2); <.001 |
| *Intensively* | 59.5 [56.4–62.5] | 49.1 [45.0–53.3] | 69.0 [65.5–72.3] |  |
| *Weekly/daily* | 37.0 [.34.0–40.1] | 45.4 [41.5–49.2] | 29.3 [26.0–32.7] |  |
| *Rarely* | 3.5 [2.9–4.3] | 5.4 [4.3–6.9] | 1.7 [1.2–2.5] |  |
| Larger friend groups |  |  |  | 0.12(2); .945 |
| *Intensively* | 34.6 [32.4-36.9] | 34.3 [31.2-37.6] | 34.9 [32.1-37.9] |  |
| *Weekly/daily* | 55.6 [53.4-57.8] | 55.9 [52.7-59.1] | 55.3 [52.3-58.3] |  |
| *Rarely* | 9.8 [8.5-11.3] | 9.8 [8.2-11.7] | 9.8 [8.1-11.7] |  |
| Online ‘friends’ |  |  |  | 0.89 (2); .666 |
| *Intensively* | 22.4 [20.5-24.3] | 21.7 [19.1-24.6] | 23.0 [20.7-25.5] |  |
| *Weekly/daily* | 47.5 [45.4-49.6] | 48.5 [45.2-51.7] | 46.5 [43.6-49.5] |  |
| *Rarely* | 30.1 [27.8-32.6] | 29.8 [26.5-33.3] | 30.5 [27.5-33.6] |  |
| Unknown people |  |  |  | 24.42(2), <.001 |
| *Intensively* | 12.9 [11.2-14.7] | 16.1 [13.7-18.8] | 9.5 [7.5-12.0] |  |
| *Weekly/daily* | 32.8 [30.6-35.1] | 34.4 [31.3-37.7] | 31.2 [28.2-34.3] |  |
| *Rarely* | 54.3 [51.6-57.0] | 49.5 [45.6-53.4] | 59.3 [55.8-62.8] |  |
| **Preference for online communication** |  |  |  |  |
| Secrets |  |  |  | 4.86(2), 0.098 |
| *Agree* | 21.9 [20.1-23.7] | 23.5 [21.2-26.0] | 20.3 [18.1-22.7] |  |
| *Neither/nor* | 26.1 [24.4-27.8] | 25.0 [22.6-27.4] | 27.1 [24.8-29.6] |  |
| *Disagree* | 52.1 [49.6-54.5] | 51.5 [48.5-54.5] | 52.6 [49.4-54.5] |  |
| Feelings |  |  |  | 15.92(2), <.001 |
| *Agree* | 27.7 [25.8-29.4] | 24.3 [22.0-26.7] | 30.9 [28.2-33.7] |  |
| *Neither/nor* | 27.0 [25.4-28.7] | 28.7 [26.5-31.0] | 25.5 [23.4-27.7] |  |
| *Disagree* | 45.3 [42.9-47.6] | 47.0 [44.2-50.0] | 43.6 [40.6-46.7] |  |
| Concerns |  |  |  | 10.77(2), .004 |
| *Agree* | 23.3 [21.5-25.2] | 20.6 [18.4-23.0] | 25.8 [23.3-28.4] |  |
| *Neither/nor* | 27.1 [25.4-28.9} | 28.1 [25.7-30.7] | 26.2 [24.1-28.4] |  |
| *Disagree* | 49.6 [47.2-52.0} | 51.3 [48.3-54.3] | 48.0 [45.0-51.1] |  |
| **Internet activity** |  |  |  |  |
| New friends |  |  |  | 67.56(2), <.001 |
| *Daily* | 10.0 [8.8-11.3] | 12.9 [11.0-15.1] | 7.3 [6.1-8.8] |  |
| *Weekly* | 23.1 [21.4-24.8] | 27.6 [25.3-30.1] | 18.9 [16.8-21.1] |  |
| *Rarely* | 66.9 [64.8-69.0] | 59.5 [56.5-62.4] | 73.8 [71.2-76.2] |  |
| Similar company |  |  |  | 30.34 (2), <.001 |
| *Daily* | 9.5 [8.4-10.6] | 11.6 [9.9-13.5] | 7.5 [6.2-9.0] |  |
| *Weekly* | 22.1 [20.5-23.8] | 24.8 [22.6-27.1] | 19.6 [17.5-21.9] |  |
| *Rarely* | 68.5 [66.5-70.3] | 63.3 [60.8-66.3] | 72.9 [70.5-75.2] |  |

**Intensive online communication**: Rarely = Never/Almost never; Weekly/daily = Every week /Daily; Intensively= Several times daily /Almost all the time.

**Preference for online communication**: Disagree = Strongly disagree & Disagree; Neither/Nor = Neither/nor; Agree = Strongly agree & Agree

**Internet activity:** Rarely = Never & Less than once a week; Weekly = Once a week & Several days a week; Daily = Every day once a day & Several times every day.

Supplement 2. Prevalence of intensive online communication, preference for online communication, and using the internet daily to look for new friends and company. Associations (Χ²) and differences (95% CI) by **age**.

|  | 11 yrs  % [95% CI] | 13 yrs  % [95% CI] | 15 yrs  % [95% CI] | Χ²(df), p-value |
| --- | --- | --- | --- | --- |
| **Intensive online communication** |  |  |  |  |
| Close friends |  |  |  | 180.04(2), <.001 |
| *Intensively* | 41.4 [34.5–45.4] | 63.5 [59.1–67.7] | 70.8 [67.8–73.6] |  |
| *Weekly/daily* | 54.6 [50.7–58.6] | 33.1 [29.0–37.5] | 26.0 [23.3–28.9] |  |
| *Rarely* | 4.0 [3.0–5.4] | 3.4 [2.3–4.9] | 3.2 [2.3–4.6] |  |
| Larger friend groups |  |  |  | 67.16(4), <.001 |
| *Intensively* | 23.8 [20.6–27.2] | 36.0 [32.5–39.6] | 42.3 [39.1–45.5] |  |
| *Weekly/daily* | 64.9 [61.4–68.2] | 54.6 [50.7–58.5] | 49.0 [46.0–51.9] |  |
| *Rarely* | 11.3 [8.5–15.0] | 9.4 [7.3–12.2] | 8.8 [7.3–10.6] |  |
| Online ‘friends’ |  |  |  | 73.96(4), <.001 |
| *Intensively* | 13,8 [10.9–17.2] | 22.5 [19.8–25.4] | 27.7 [25.1–30.5] |  |
| *Weekly/daily* | 42.9 [38.9–47.1] | 49.9 [46.7–53.2] | 48.2 [44.6–51.8] |  |
| *Rarely* | 43.3 [39.3–47.4] | 27.6 [24.1–31.3] | 24.1 [20.8–27.7] |  |
| Unknown people |  |  |  | 57.09(4), <.001 |
| *Intensively* | 8.5 [6.0–12.0] | 14.3 [11.5–17.6] | 12.9 [11.2–14.8] |  |
| *Weekly/daily* | 20.7 [17.9–23.9] | 33.0 [29.5–36.8] | 38.7 [35.4–42.1] |  |
| *Rarely* | 70.7 [66.7–74.5] | 52.7 [48.7–56.7] | 47.7 [43.6–51.3] |  |
| **Preference for online communication** |  |  |  |  |
| Secrets |  |  |  | 88.09(4), <.001 |
| *Agree* | 13.3 [11.1–15.9] | 24.0 [21.6–26.7] | 27.0 [24.2–30.0] |  |
| *Neither/nor* | 22.1 [18.9–25.7] | 27.9 [25.1–30.8] | 27.7 [25.4–30.1] |  |
| *Disagree* | 64.6 [60.7–68.3] | 48.1 [44.8–51.5] | 45.4 [42.1–48.7] |  |
| Feelings |  |  |  | 103.13(4), <.001 |
| *Agree* | 17.3 [14.8–20.1] | 31.2 [28.5–36.4] | 27.7 [25.7–29.7] |  |
| *Neither/nor* | 23.9 [21.2–26.9] | 28.1 [25.4–30.9] | 28.7 [26.2–31.4] |  |
| *Disagree* | 58.8 [55.4–62.2] | 40.7 [37.7–43.8] | 38.3 [35.2–41.6] |  |
| Concerns |  |  |  | 107.46(4), <.001 |
| *Agree* | 13.5 [11.1–16.3] | 25.5 [23.2–27.9] | 29.6 [26.8–32.5] |  |
| *Neither/nor* | 23.4 [20.3–26.8] | 29.1 [26.1–32.2] | 28.4 [26.1–30.8] |  |
| *Disagree* | 63.1 [59.5–66.7] | 45.5 [42.2–48.8] | 42.1 [38.8–45.4] |  |
| **Internet activity** |  |  |  |  |
| New friends |  |  |  | 64.5(4), <.001 |
| *Daily* | 6.4 [5.0–8.1] | 10.8 [9.1–12.8] | 12.2 [10.0–14.7] |  |
| *Weekly* | 77.2 [73.8–80.4] | 65.1 [61.8–68.3] | 59.9 [56.8–63.0] |  |
| *Rarely* | 16.4 [13.7–19.5] | 24.0 [21.6–26.6] | 27.9 [25.1–30.8] |  |
| Similar company |  |  |  | 31.35 (4), <.001 |
| *Daily* | 6.8 [5.4–8.4] | 9.9 [8.3–11.6] | 11.3 [9.3–13.8] |  |
| *Weekly* | 17.5 [14.8–20.6] | 23.8 [21.4–26.4] | 24.3 [21.6–27.1] |  |
| *Rarely* | 75.7 [72.7–78.6] | 66.3 [63.5–69.1] | 64.4 [60.8–67.9] |  |

**Intensive online communication**: Rarely = Never/Almost never; Weekly/daily = Every week /Daily; Intensively= Several times daily /Almost all the time.

**Preference for online communication**: Disagree = Strongly disagree & Disagree; Neither/Nor = Neither/nor; Agree = Strongly agree & Agree

**Internet activity:** Rarely = Never & Less than once a week; Weekly = Once a week & Several days a week; Daily = Every day once a day & Several times every day.

Supplement 3. Prevalence of intensive online communication, preference for online communication, and using the internet daily to look for new friends and company. Associations (Χ²) and differences (95% CI) by **HL level**.

|  | Low  % [95% CI] | Moderate  % [95% CI] | High  % [95% CI] | Χ²(df), p-value |
| --- | --- | --- | --- | --- |
| **Intensive online communication** |  |  |  |  |
| Close friends |  |  |  | 37.07(4). <.001 |
| *Intensively* | 59.9 [50.0–69.0] | 64.7 [61.2–68.1] | 73.1 [69.3–76.7] |  |
| *Weekly/daily* | 31.5 [23.2–41.2] | 31.6 [28.4–35.0] | 25.8 [22.4–29.6] |  |
| *Rarely* | 8.6 [5.3–13.8] | 3.7 [2.6–5.2] | 1.0 [0.5–2.1] |  |
| Larger friend groups |  |  |  | 42.73(4), <.001 |
| *Intensively* | 30.8 [23.7–38.9] | 36.7 [33.5–40.1] | 44.9 [40.8–49.1] |  |
| *Weekly/daily* | 50.6 [43.0–58.3] | 53.3 [50.0–56.6] | 50.5 [46.6–54.5] |  |
| *Rarely* | 18.6 [13.9–24.4] | 10.0 [8.1–12.3] | 4.6 [3.2–6.5] |  |
| Online ‘friends’ |  |  |  | 4.13(4), 0.470 |
| *Intensively* | 29.7 [21.5–39.4] | 23.9 [21.3–39.4] | 25.2 [23.2–27.3] |  |
| *Weekly/daily* | 43.8 [34.5–53.5] | 51.0 [47.7–54.3] | 49.0 [46.6–51.5] |  |
| *Rarely* | 26.6 [19.0–35.7] | 25.1 [21.9–28.6] | 25.8 [23.4–28.3] |  |
| Unknown people |  |  |  | 12.68(4), 0.018 |
| *Intensively* | 15.8 [9.25–25.6] | 12.1 [10.0–14.7] | 16.4 [13.1–20.3] |  |
| *Weekly/daily* | 46.5 [38.1–55.1] | 36.7 [32.9–40.6] | 32.7 [29.4–36.3] |  |
| *Rarely* | 37.7 [29.3–46.9] | 51.2 [47.2–55.2] | 50.1 [46.2–55.6] |  |
| **Preference for online communication** |  |  |  |  |
| Secrets |  |  |  | 28.96(4), <.001 |
| *Agree* | 33.0 [26.6–39.9] | 25.6 [23.1–28.2] | 23.3 [20.0–26.8] |  |
| *Neither/nor* | 25.8 [20.5–31.9] | 31.5 [29.2–34.0] | 22.8 [19.7–26.3] |  |
| *Disagree* | 41.3 [34.3–48.7] | 42.9 [40.1–45.7] | 54.0 [50.1–57.8] |  |
| Feelings |  |  |  | 29.18(4), <.001 |
| *Agree* | 36.5 [29.0–44.8] | 33.1 [30.0–36.3] | 29.0 [25.4–32.9] |  |
| *Neither/nor* | 25.8 [20.3–32.0] | 32.1 [29.5–34.7] | 28.7 [26.8–30.7] |  |
| *Disagree* | 37.7 [30.5–45.6] | 34.9 [32.3–37.6] | 47.0 [37.2–41.7] |  |
| Concerns |  |  |  | 32.11(4), <.001 |
| *Agree* | 34.1 [27.9–40.9] | 27.4 [24.6–30.3] | 25.7 [22.5–29.2] |  |
| *Neither/nor* | 28.7 [22.0–36.6] | 32.8 [30.2–35.5] | 22.9 [19.7–26.5] |  |
| *Disagree* | 37.1 [30.1–44.7] | 39.9 [36.9–42.9] | 51.4 [47.2–55.5] |  |
| **Internet activity** |  |  |  |  |
| New friends |  |  |  | 7.84(4), .120 |
| *Daily* | 12.0 [7.4–18.9] | 10.0 [8.2–12.2] | 13.4 [10.9–16.3] |  |
| *Weekly* | 31.1 [25.6–37.3] | 26.3 [23.4–29.4] | 24.3 [21.4–27.5] |  |
| *Rarely* | 56.9 [49.4–64.1] | 63.7 [60.1–67.1] | 62.3 [58.5–65.9] |  |
| Similar company |  |  |  | 4.45(4), .394 |
| *Daily* | 13.1 [8.2–20.4] | 9.4 [7.9–11.1] | 12.0 [9.5–15.0] |  |
| *Weekly* | 25.0 [18.8–32.5] | 24.2 [21.7–26.8] | 23.3 [20.4–26.5] |  |
| *Rarely* | 61.9 [52.4–70.6] | 66.5 [63.5–69.3] | 64.8 [61.1–68.2] |  |

**Intensive online communication**: Rarely = Never/Almost never; Weekly/daily = Every week /Daily; Intensively= Several times daily /Almost all the time.

**Preference for online communication**: Disagree = Strongly disagree & Disagree; Neither/Nor = Neither/nor; Agree = Strongly agree & Agree

**Internet activity:** Rarely = Never & Less than once a week; Weekly = Once a week & Several days a week; Daily = Every day once a day & Several times every day.

Supplement 4. Correlations between latent online communication variables; correlations between well-being indicators.

| **Latent factors for online communication** | **OC1** | **OC2** | **OC3** |  |
| --- | --- | --- | --- | --- |
| Intensive online communication with friends (OC1) | - |  |  |  |
| Intensive online communication with people one has only met online (OC2) | .64*** | - |  |  |
| Preference for online communication in personal matters (OC3) | .16*** | .34 *** | - |  |
| Company seeking behaviour on the Internet (OC4) | .33*** | .62*** | .28*** |  |
|  |  |  |  |  |
| **Well-being indicators** | **W1** | **W2** | **W3** | **W4** |
| Loneliness (W1) | - |  |  |  |
| Cyberbullying victimization (W2) | .23*** |  |  |  |
| Self-rated health (W3) | -.25*** | -.15*** |  |  |
| Life satisfaction (W4) | -.42*** | -.19*** | .42*** |  |
| Problematic social media use (W5) | .19*** | .12*** | -.13*** | -.17*** |
